# Preclinical research performed on reanimated/perfused swine kidneys: The Visible Kidney™ methodologies

**DOI:** 10.14814/phy2.15630

**Published:** 2023-03-06

**Authors:** Thomas F. Valenzuela, Emma Schinstock, Samantha Kohnle, Azeem Latib, Dimitrios Bliagos, Stefan Tunev, Paul A. Iaizzo

**Affiliations:** ^1^ Medtronic Minneapolis Minnesota USA; ^2^ The Department of Surgery's Visible Heart® Laboratories and the Institute for Engineering in Medicine University of Minnesota Minneapolis Minnesota USA; ^3^ Montefiore Medical Center, Albert Einstein College of Medicine The Bronx New York USA

## Abstract

Preclinical research remains the essential platform in the development and optimization of medical therapies and advancements in translational medicines. However, specifically to animal research, federal laws, and institutional policies require investigators to apply the principles of the 3R's (replacement, reduction, and refinement). The concept of benchtop models utilizing isolated organs, in which multiple variables can be controlled to recreate human function, has been innovative advancements in preclinical research models that adhere to these principles. More specifically, isolated perfused kidney (IPK) models have been invaluable preclinical tools that have led to numerous advancements over the decades, including understanding renal physiology, pharmacologic therapies, and improvements in renal transplantation. However, pre‐existing IPK models are not without their own limitations, leaving areas for improvement. An isolated perfused kidney apparatus was designed to best recreate human use conditions as a preclinical tool. Porcine renal blocks were chosen over the more commonly used rodent models, due to their greater similarities to human anatomies. Sixteen porcine kidney pairs obtained en bloc were extracted and placed onto an apparatus where aortic flows, pressures, and overall systemic temperatures were controlled. Organ viability was assessed in 10 renal blocks (*n* = 8 fresh and *n* = 2 previously frozen specimens) via both urinary flows and compositions at timepoints up to 180 min. Multimodality imaging, which included fluoroscopy, ultrasound, optical coherence tomography (OCT), and video scopes, was also employed to capture internal and external images to determine renal artery orientations and dimensions. Anatomical measurements and viability assessments of porcine renal blocks were successfully achieved in our perfusion model. Renal main artery diameters averaged smaller in our sample size than in human anatomy while also having more superior takeoff angles. Yet, the average lengths of each main segment were comparable to human anatomy: 32.09 ± 7.97 mm and 42.23 ± 7.33 mm in the left and right renal main artery, respectively. Urine production and urine composition of the fresh renal blocks, when compared to the frozen blocks and baseline perfusate, showed kidney viabilities of up to 3 h via excretion and retention of various metabolites. In this paper, we described a protocol for an isolated perfused kidney apparatus using large mammalian renal blocks. We believe this protocol to be an improvement from similar pre‐existing models in better representing human physiologic function while allowing for multimodal imaging. The resulting Visible Kidney™ preclinical model, which has shown viability after isolation and reperfusion, can be a fast and reliable tool for the development of medical devices while also reducing the unnecessary use of animals for research.

## Graphical abstract

Isolated perfused kidney (IPK) models have been invaluable preclinical tools. However, they are still not without their own limitations. We believe the methodology described in this manuscript to be an improvement on pre‐existing IPK models that better recreates human use conditions while showing organ viability.

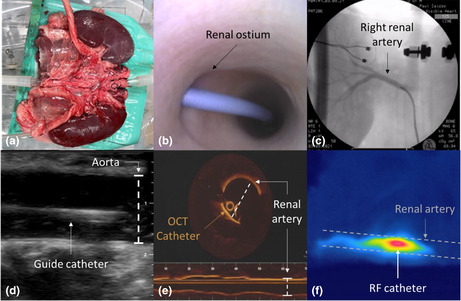

## 1 INTRODUCTION

Preclinical research remains as an essential platform in the development and optimization of medical devices, therapies, and advancements in translational medicines. Preclinical research is typically performed on animal subjects to ensure its safety and efficacy before it is trialed in humans. However, federal laws and institutions have put forth policies like the Animal Welfare Act (AWA) which regulates the care, treatment, and use of animals in research. Such policies require investigators to apply the principles of the 3Rs (replacement, reduction, and refinement) when conducting animal research (USDA, n.d.; Mandal & Parija, 2013). Innovative advancements in preclinical research models, such as the use of isolated organs in controlled environments to recreate human physiologic functions, have enabled for the continuous development of devices, medicines, and therapies while adhering to these principles.

The concept of research utilizing an isolated perfused kidney (IPK) first started in the early 20th century (Hemingway & Schweitzer, 1944; Macgregor & Peat, 1933). Various iterations of the IPK model have allowed for great advancements to be made in fields such as renal physiology, pharmacology, and transplantations (Aiba et al., 1999; Ciarimboli et al., 2003; Cupples & Loutzenhiser, 1998; Epstein, 1991; Georgiev et al., 2011; Hemingway & Schweitzer, 1944; Höchel et al., 2003; Macgregor & Peat, 1933; Nishiitsutsuji‐Uwo et al., 1967; Quilley et al., 1993; Unger et al., 2007; Weiss et al., 1959). While the first experiments were performed on organs from larger animals such as cats or dogs (Hemingway & Schweitzer, 1944; Höchel et al., 2003; Macgregor & Peat, 1933), most modern studies of renal function have scaled down and have moved onto the IPK rat model (Aiba et al., 1999; Ciarimboli et al., 2003; Cupples & Loutzenhiser, 1998; Epstein, 1991; Nishiitsutsuji‐Uwo et al., 1967; Quilley et al., 1993; Weiss et al., 1959). The smaller rat model may be a preferred model for some pharmacological researchers due to the physiological and morphological similarities (Georgiev et al., 2011) and their smaller sizes and lower costs.

There are various forms of the IPK models, such as single or double kidney in vitro and in situ models (Boesen & Pollock, 2007; Cox et al., 1991; Schneider et al., 2007) which aim to reduce the total number of animals used. Some of these models also extend into total replacement using silicone models (Carl von et al., 2017). However, despite the reduction and replacement in the larger animal IPK model, there still exists physiologic and morphologic differences when using smaller mammals, especially silicone models, and real human renal characteristics. A larger mammalian model, like the porcine model, may be preferred because of the greater similarities with human renal anatomy. This is also critical for the development of clinically employed ex vivo perfusion technologies. Such IPK models need to be developed to better recreate human physiology and use conditions to improve our understanding of device‐tissue interactions, clinical translation, and physician training.

We have sought to develop a pre‐clinical research tool for IPKs based off methodologies utilized for reanimating large mammalian hearts, including human, employing the Visible Heart® Apparatus (Chinchoy et al., 2000). For two and a half decades, the University of Minnesota's Visible Heart® Laboratories has incorporated multimodal imaging of these functioning hearts sustained with a clear perfusate. By using organ donor tissues acquired via an organ procurement organization, LifeSource, and performing evaluations and experiments on these specimens under replicated physiological conditions, our research team has been able to refine, reduce, and occasionally replace the use of animals for such cardiac research (Bateman et al., 2020; Holm et al., 2020; Iles et al., 2020; Mattson et al., 2019; Zhingre Sanchez et al., 2019); which has been critical for both cardiac device development and physician training. In the present study, we described the development and utilization of a pre‐clinical Visible Kidney™ apparatus for the same aforementioned purposes.

## 2 MATERIALS AND METHODS

Organs were first isolated and prepared for reanimation, then placed on the developed apparatus, then imaged, measured, and evaluated for viability over a period of 3 h.

### 2.1 Animal preparation and organ isolation

Healthy male Yorkshire swine (80–90 kg) were used following approval from the University of Minnesota's Animal Care and Use Committee. Telazol (≤500 mg/kg) was administered intramuscularly, and intravenous access was obtained through an ear vein for volume administration and delivery of Methohexital (≤50 mg/kg) and other medication(s) as needed. The animal was then intubated, and isoflurane was continuously administered to maintain a 1‐to‐1.5 minimum alveolar concentration (MAC). Once under a full plane of anesthesia, a medial sternotomy was performed to access the heart to allow for a standard cardioplegia protocol using a St. Thomas' Hospital cardioplegic solution (NaCl 110.0 mM, NaHCO_3_ 10.0 mM, KCl 16.0 mM, MgCl_2_ 16.0 mM, CaCl_2_ 1.2 mM, pH 7.8). Immediately postheart recovery, a laparotomy was performed to expose the abdominal cavity and abdominal organs. The peritoneal lining, and all the organs within, were carefully dissected away to access the retroperitoneal space. From there, a tissue block including the dorsal muscles, aorta, inferior vena cava (IVC), bilateral kidneys, renal arteries, renal veins, and ureters were extracted to ensure anatomical integrity. Once extracted, the kidney block was carefully prepared for isolated ex vivo perfusion.

### 2.2 Recreating physiological use conditions in an ex vivo apparatus

**FIGURE 1:**
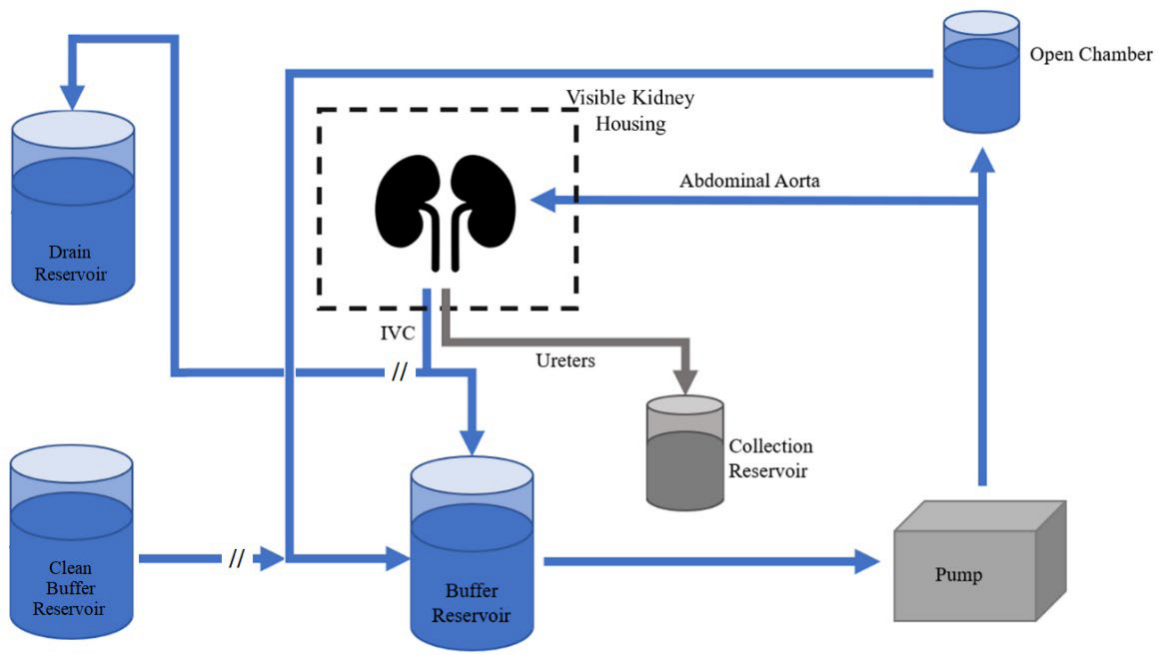
Diagram of the ex vivo kidney perfusion setup. The buffer leaves the pump and is directed upward where the flow is split toward the elevated open chamber, which regulates the pressure, and the abdominal aorta. Flow through the aorta is directed through the renal arteries, circulated within the kidneys, and returned through the IVC so that it could be recirculated into the buffer reservoir, whereas the fluid from each ureter was collected separately.

Once cannulated, the kidney block was placed in a modified OCS™ Lung housing capsule (TransMedics Inc.) to best represent the human abdominal cavity. The clear polycarbonate enclosure was removed from the original OCS cartridge and altered to maintain the kidneys in an anatomical position while allowing for inflow, outflow, and sampling lines to pass in and out of the housing. Once placed in the housing, the specimen was attached to the perfusion apparatus, Figure 1, and the casing was closed prior to perfusion. Through pilot studies, it was determined that to best recreate human use conditions using an IPK, the specimen must be exposed to a representative normothermic microenvironment. This meant placing the IPK within an enclosed case where temperature and relative humidity of the capsule can be controlled to best recreate the human abdomen. This was achieved using an infrared (IR) bulb located above the capsule, and a warming pad placed underneath the tissue block in addition to the buffer that was circulated throughout the anatomy.

A modified Krebs Henseleit buffer, heated to 37°C, with direct oxygenation using carbogen (95% O_2_, 5% CO_2_) was used throughout these ex vivo experiments. This clear perfusate was delivered via a pulsatile pump (Harvard Apparatus) that branched off into an adjustable column, to allow for independent pressure control, and through the cannulated abdominal aorta which allowed flow into the renal arteries at a rate of 60 beats per minute (bpm). Stroke volume was adjusted appropriately per donor based on their relative size, so that perfusate flow between both renal arteries was within normal human ranges, ~1 L/min (Prowle et al., 2009). Next, the pressure column was adjusted to a height where the internal anatomical pressure had a systolic peak of 120 mm Hg.

Flow delivered through the renal arteries was circulated through the renal microvasculature and through the renal venous system back toward the inferior vena cava and a portion was excreted via the ureters, which was not recirculated. Catheterization of the ureters was performed so that the produced urine was filtered from the circulating buffer and collected separately. The vena cava was not cannulated to allow for the perfusate to flow out into a reservoir where it is recirculated back into the main buffer source (the TransMedic approach).

### 2.3 Imaging

**FIGURE 2:**
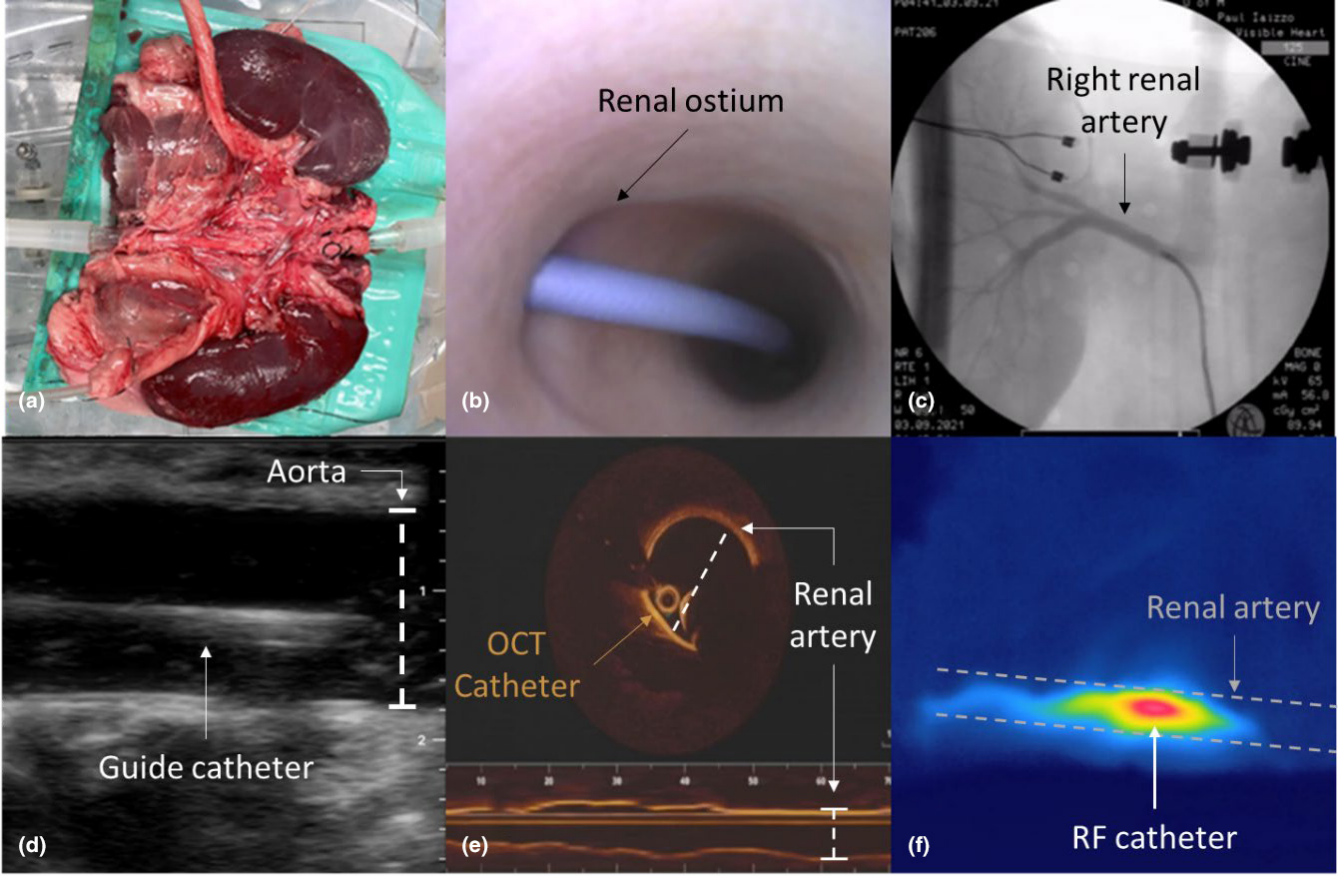
Once placed within the perfusion apparatus, (a) overhead cameras are used to visualize the external surface of the anatomy. (b) Small bore endoscopic cameras are inserted within the system to visualize the internal anatomy. Shown here is the introduction of a 6F guide catheter in the right renal artery. (c) Simultaneous fluoroscopic imaging is used to capture renal angiograms and procedural imaging. (d) Ultrasound and (e) OCT are intraprocedural imaging tools that can also be used to evaluate renal arteries and/or measure renal artery flow. (f) Thermal imaging can be used to image thermal profiles during studies such as this one in which a 5F Mariner RF catheter (Medtronic Inc.) was used to perform a RF ablation.

Direct visualization utilizing overhead video cameras monitored relative renal positioning and global function (e.g., ischemia and edema). Both 2.4 and 4.0 mm in diameter endoscopic cameras (Olympus) were used to visualize and capture the internal functional anatomies of the isolated kidneys with capabilities for simultaneous fluoroscopic visualization (Ziehm Imaging). Additional multimodality imaging was also performed on the IPK such as thermal (Teledyne FLIR, Wilsonville, OR) and ultrasound imaging (Butterfly Network). Optical Coherence Tomography (OCT) utilizing OPTIS Intravascular Imaging System and Dragonfly™ Imaging Catheters (Abbott Vascular) was also used as an additional means of imaging the anatomic features of the renal vasculature, which also allowed for intraprocedural measurements to be taken. All these visual modalities were continuously recorded using a custom multichannel digital recording system (Z Systems, Inc.) as shown in Figure 2.

### 2.4 Acquiring measurements

The weight of each animal was acquired when sedated, after administrations of Telazol and Methohexatol and prior to intubation. Once intubated, arterial pressures were monitored while under anesthesia to ensure that there were no adverse cardiac events prior to organ explantation.

Once isolated and perfused, perfusate flow through the abdominal aorta and renal arteries was delivered via the pulsatile pump and the arterial pressure of the given kidney block was acquired using pressure transducers on a custom EMKA system (EMKA Technologies) to ensure an average systolic pressure of 120 mm Hg. Temperature of the kidney capsule was monitored using a temperature probe (Fluke Corporation) and maintained at 37 ± 0.5°C. Once perfused, urinary flow was measured every 15 min for up to 3 h, using a graduated cylinder and a stopwatch. Concurrently, urinary and perfusate samples were taken and analyzed using an ABL90 Flex Plus blood gas analyzer (Radiometer) to compare urine and perfusate compositions.

**FIGURE 3:**
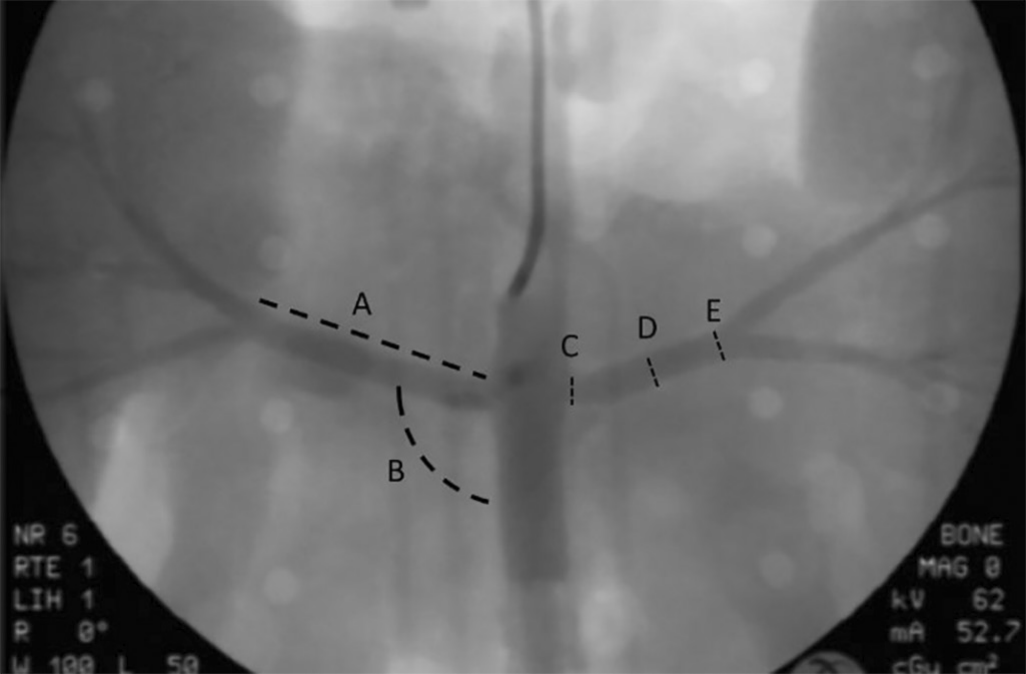
Renal angiograms taken at the start of each study were saved and then later used to obtain measurements of the (A) length of the renal main, (B) takeoff angle from the aorta and (C) proximal, (D) mid, and (E) distal main segments.

While most of the data were acquired in real time, dimensions of the renal arteries were postprocessed from renal angiograms. Images of the left and right renal angiograms were taken immediately after perfusion was started using an Isovue® contrast agent. The images were imported into ImageJ (National Institutes of Health) where the 6F (2 mm diameter) guide catheter used to deliver the contrast was used as a reference to calibrate the measuring tool. Once calibrated, the measuring tool was used to measure the length and takeoff angles of the main renal arteries as well as the diameter of the proximal, middle, and distal main segments (see Figure 3). All statistical analysis for this study was performed via Minitab software (State College).

## 3 RESULTS

An external apparatus for the perfusion of large mammalian renal blocks was created in which physiologic conditions such as simulated heart rates, flow rates, pressures, humidity, and temperatures could be controlled. Sixteen renal blocks from animals that were already slated for cardiothoracic research were used in this study. All 16 were used for the purpose of renal characterizations (*n* = 16) and 10 for viability assessments comparing fresh (*n* = 8) versus previously frozen (*n* = 2) renal blocks. For the fresh specimens, the average time between the acquisition of the tissues and when they were brought back up to physiologic temperature (37 ± 0.5°C) after reperfusion on the apparatus was 45 min. For the frozen specimens, once acquired from the animal, they were placed in a freezer for 5 days and were then thawed and re‐perfused until they were brought back up to the same physiologic temperature.

**TABLE 1:** Average porcine renal anatomical characteristics.

Parameter	Value			
Animal weight (kg)	87.40 ± 7.98 (*n* = 16)			
	LMRA		RMRA	
Artery	Value	*n*	Value	*n*
Length (mm)	32.09 ± 7.97	15	42.23 ± 7.33	16
Prox mean diam (mm)	5.18 ± 1.08	15	5.13 ± 1.00	16
Mid mean diam (mm)	4.45 ± 0.66	15	4.57 ± 0.76	16
Distal mean diam (mm)	4.53 ± 0.64	15	4.58 ± 1.01	16
Takeoff angle (°)	109.97 ± 9.62	15	100.78 ± 7.46	16

Abbreviations: LMRA, left main renal artery; RMRA, right main renal artery.

Renal angiograms were performed to measure the lengths, diameters, and takeoff angles of the main renal arteries to determine anatomical characteristics. It is worth noting that during these renal angiograms, it was discovered that one specimen of the 16 (6%) had two main renal arteries (MRA) that branched off the aorta and into the left kidney. Since this occurrence was anomalous in our study, it was excluded from the analysis. Table 1 shows the average animal size and main renal artery lengths, takeoff angles, and proximal, middle, and distal diameters.

**TABLE 2:** Urine composition averages of fresh and frozen specimens over time.

	Tissue	*t* = 0	*t* = 15	*t* = 30	*t* = 45	*t* = 60	*t* = 75	*t* = 90	*t* = 105	*t* = 120	*t* = 135	*t* = 150	*t* = 165	*t* = 180	*p*‐value
Urine rate (mL/h)	Fresh	131.5 ± 64.7	122.6 ± 59.0	142.9 ± 41.7	144.8 ± 29.7	154.8 ± 49.3	143.6 ± 42.1	141.5 ± 44.5	130.9 ± 37.6	120.0 ± 42.0	112.1 ± 41.8	108.4 ± 40.4	103.0 ± 35.0	97.3 ± 24.2	Time 0.245
	Frozen	338.5 ± 87.0	1475.0 ± 1237.0	1540.0 ± 84.9	1725.0 ± 318.0	1527.0 ± 401.0	1355.0 ± 453.0	1055.0 ± 453.0	1028.0 ± 491.0	990.0 ± 226.0	910.0 ± 191.0	866.5 ± 120.9	777.0 ± 39.6	862.5 ± 91.2	Tissue 0.000
pH	Fresh	6.95 ± 0.05	6.95 ± 0.14	6.91 ± 0.15	6.91 ± 0.15	6.94 ± 0.16	6.93 ± 0.15	6.90 ± 0.15	6.91 ± 0.12	6.92 ± 0.14	6.87 ± 0.13	6.87 ± 0.12	6.84 ± 0.15	6.82 ± 0.13	Time 0.266
	Frozen	7.37 ± 0.33	7.64 ± 0.05	7.69 ± 0.06	7.70 ± 0.02	7.74 ± 0.05	7.75 ± 0.02	7.75 ± 0.04	7.77 ± 0.04	7.78 ± 0.01	7.78 ± 0.01	7.78 ± 0.01	7.77 ± 0.02	7.77 ± 0.03	Tissue 0.000
cNa (mmol/L)	Fresh	123.75 ± 2.31	124.63 ± 1.85	124.00 ± 3.12	124.50 ± 3.42	125.13 ± 4.22	125.43 ± 4.16	125.57 ± 4.50	126.83 ± 1.72	127.33 ± 1.51	126.50 ± 1.05	126.00 ± 1.79	125.67 ± 2.50	124.43 ± 3.99	Time 0.033
	Frozen	126.50 ± 4.95	128.00 ± 4.24	127.50 ± 4.95	128.00 ± 4.24	128.00 ± 4.24	128.00 ± 4.24	128.50 ± 4.95	128.50 ± 4.95	128.50 ± 4.95	129.00 ± 4.24	129.00 ± 4.24	129.00 ± 4.24	129.00 ± 4.24	Tissue 0.000
cK (mmol/L)	Fresh	7.83 ± 1.32	6.19 ± 0.91	5.66 ± 0.72	5.26 ± 0.48	5.00 ± 0.39	4.83 ± 0.39	4.76 ± 0.33	4.66 ± 0.33	4.74 ± 0.35	4.78 ± 0.39	4.79 ± 0.36	4.81 ± 0.35	4.84 ± 0.43	Time 0.000
	Frozen	5.15 ± 0.78	4.40 ± 0.28	4.75 ± 0.07	4.70 ± 0.00	4.65 ± 0.07	4.50 ± 0.00	4.50 ± 0.00	4.55 ± 0.07	4.55 ± 0.07	4.45 ± 0.07	4.40 ± 0.14	4.40 ± 0.00	4.35 ± 0.07	Tissue 0.000
cCa (mg/dL)	Fresh	5.33 ± 0.59	5.35 ± 0.50	5.21 ± 0.45	5.13 ± 0.45	5.14 ± 0.46	5.15 ± 0.46	5.18 ± 0.48	5.21 ± 0.48	5.25 ± 0.47	5.25 ± 0.47	5.24 ± 0.48	5.23 ± 0.49	5.23 ± 0.51	Time 0.745
	Frozen	4.43 ± 0.14	4.60 ± 0.07	4.52 ± 0.00	4.52 ± 0.01	4.52 ± 0.05	4.60 ± 0.11	4.58 ± 0.10	4.55 ± 0.08	4.52 ± 0.09	4.52 ± 0.08	4.51 ± 0.04	4.49 ± 0.12	4.38 ± 0.06	Tissue 0.000
cGluc (mg/dL)	Fresh	168.75 ± 4.56	168.88 ± 5.19	168.38 ± 7.63	168.50 ± 7.13	168.88 ± 6.71	170.38 ± 7.91	171.63 ± 8.48	172.75 ± 8.83	173.63 ± 7.42	172.50 ± 9.20	173.50 ± 9.06	172.75 ± 10.62	171.63 ± 9.01	Time 0.051
	Frozen	176.50 ± 10.61	183.00 ± 5.66	182.50 ± 7.78	183.00 ± 7.07	184.00 ± 7.07	183.00 ± 8.49	183.00 ± 8.49	181.50 ± 7.78	180.50 ± 4.95	180.00 ± 4.24	180.50 ± 4.95	179.50 ± 4.95	180.50 ± 0.71	Tissue 0.000

**FIGURE 4:**
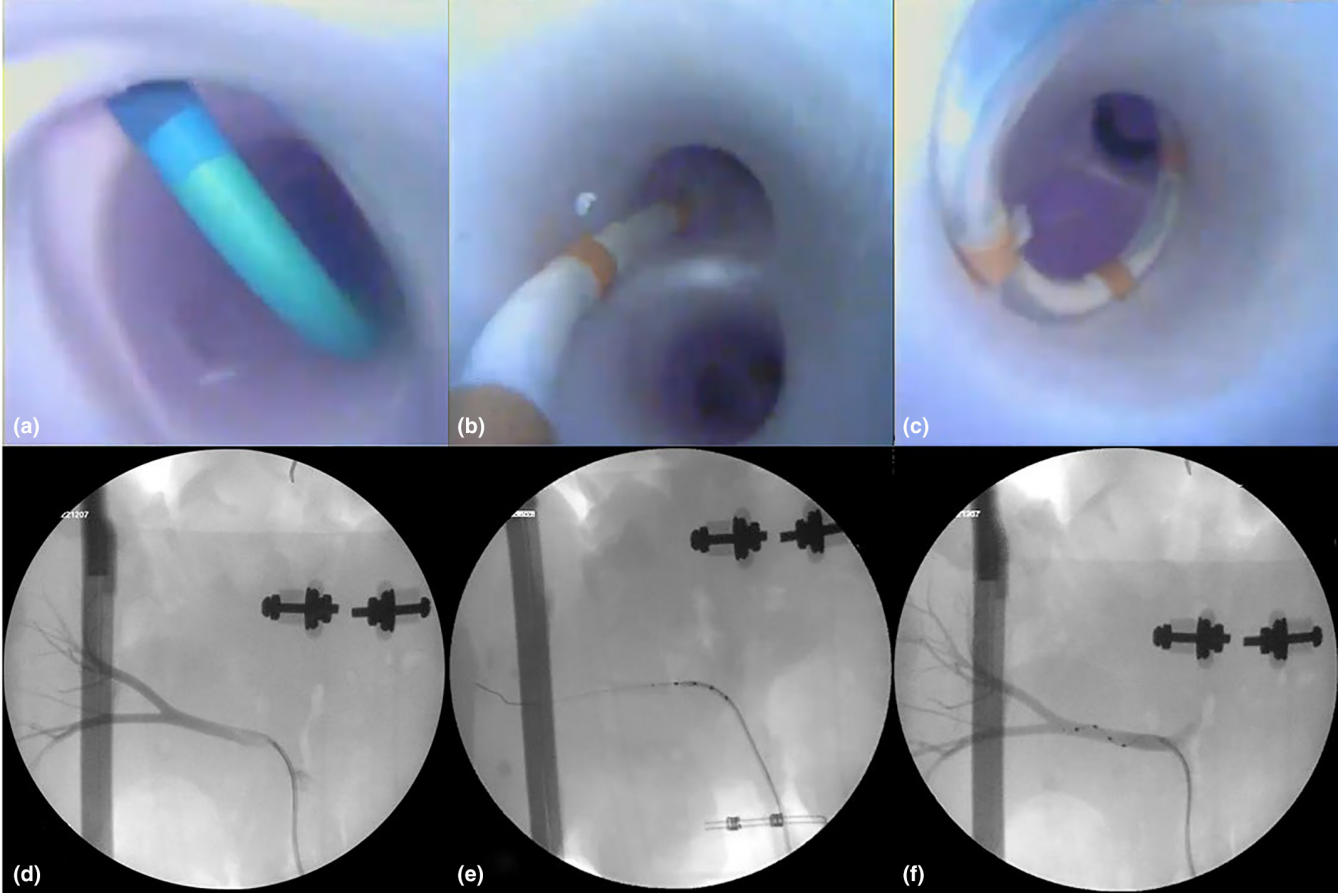
Due to the multimodal imaging capability of this IPK model, endoscopic cameras and fluoroscopy can be used simultaneously to visualize a guide catheter gaining access to a renal artery (a, d), deliver (b, e) and deploy (c, f) a Symplicity Spyral™ catheter.

Visualization of the internal anatomy via direct endoscopic imaging was made possible due to the use of the clear Krebs Henseleit buffer. This allowed for multimodal imaging of medical devices from both direct and clinical imaging to be used for the observation of device‐tissue interactions. For example, Figure 4 shows the delivery and deployment of a Symplicity Spyral™ catheter (Medtronic Inc.) from both direct and fluoroscopic imaging.

**FIGURE 5:**
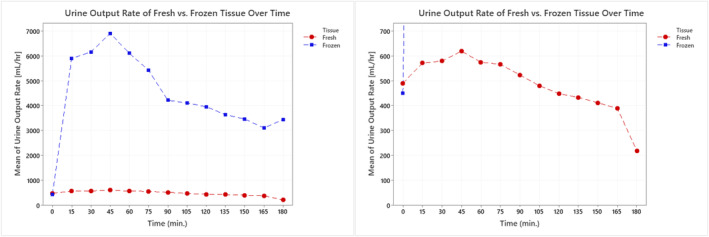
(Left) Average urine output rates varied between the fresh and frozen renal blocks with the frozen renal blocks having significantly higher rates. (Right) When reducing the scale to better look at urine output rates of the fresh specimens, similar trends were noted between the varying conditions.

Viability assessments were performed in 10 of the 16 specimens, once the protocol and methods for determining viability were finalized. During these assessments, urine volume being excreted from each ureter was monitored to indicate acute renal function. Total urine output (mL/h) was estimated every 15 min throughout each 3‐h study. Figure 5 below shows the average urine output for both the fresh and frozen specimens.

**FIGURE 6:**
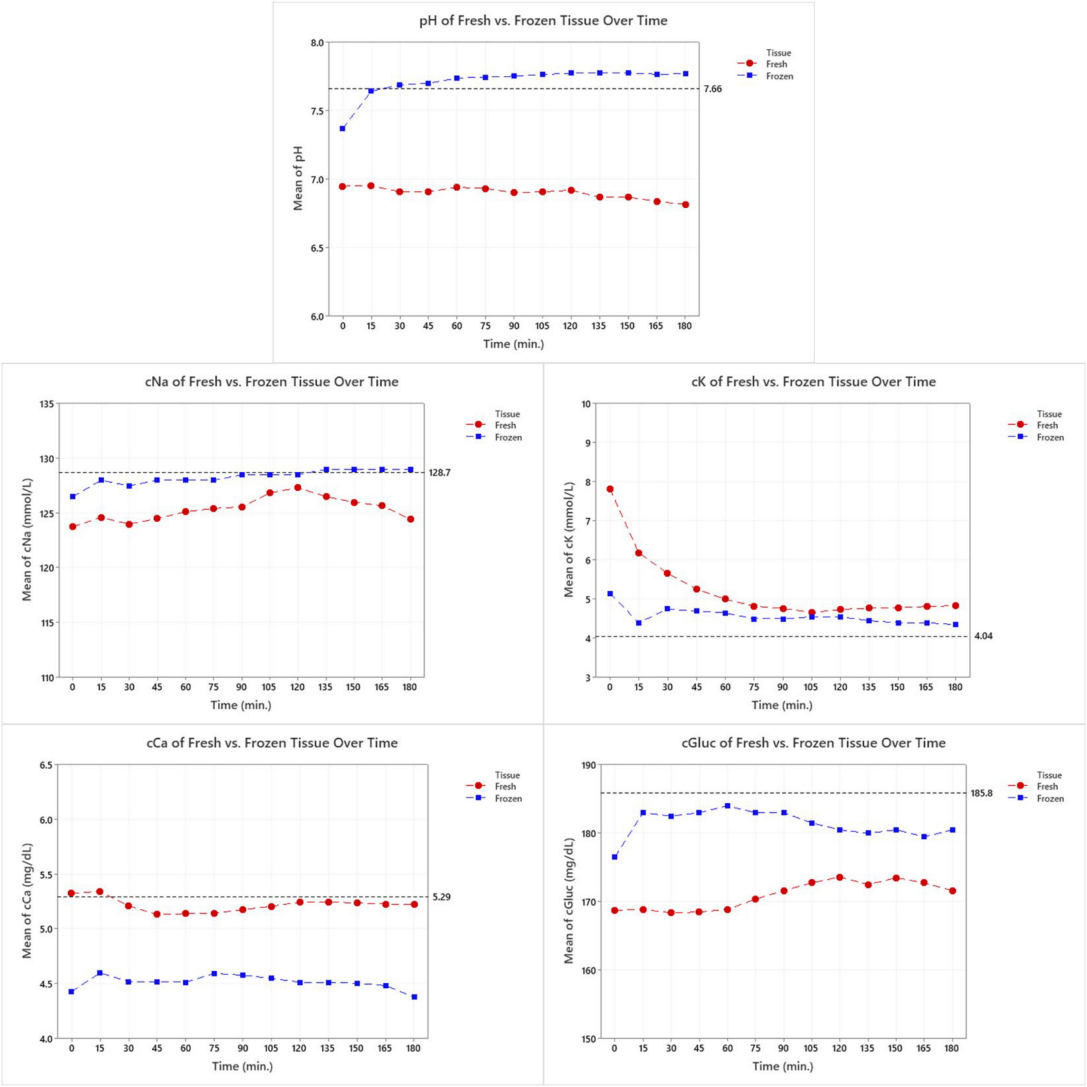
Presented here is a graphic representation of the average values for the pH and specific components of the urine measured from fresh (red) and frozen (blue) specimens at each time interval. The average value of the pH and specific components of the Krebs buffer across all 10 studies is displayed as the black dotted lines.

After the collection and measurement of the urine sample at each time interval, an aliquot was taken from both the urine and the circulating Krebs buffer and analyzed in real time to measure and compare the pH and concentration of sodium, potassium, chloride, and glucose amounts of the excreted urine to the perfusate. Figure 6 shows the average pH and concentrations of each aliquot from the excreted urine throughout all eight viability studies. The black reference lines in each graph show the average concentration of the same chemicals in the Krebs solution at each time point across all 10 studies (*n* = 10). The averages of all measurements taken at each time point from both specimens are shown in Table 2. Additionally, a multivariate analysis of variance (MANOVA) was performed for each measured output to assess the differences in means caused over time and by tissue types.

## 4 DISCUSSION

In developing the Visible Kidney™ preclinical benchtop model that takes into consideration physiologic human use conditions, we believe to have enhanced the use of large mammalian IPK models. The use of this model for pre‐clinical testing allows researchers to reduce the costs and perhaps lower the number of animals needed to effectively evaluate a treatment or therapy. Since we have shown isolated kidney viability for up to 3 h, multiple experiments can be performed on an IPK from a single donor. Furthermore, even if the proposed experiments are destructive or anatomy altering in nature, researchers can perform them after concluding nondestructive experimentations, while the organs are still viable.

**FIGURE 7:**
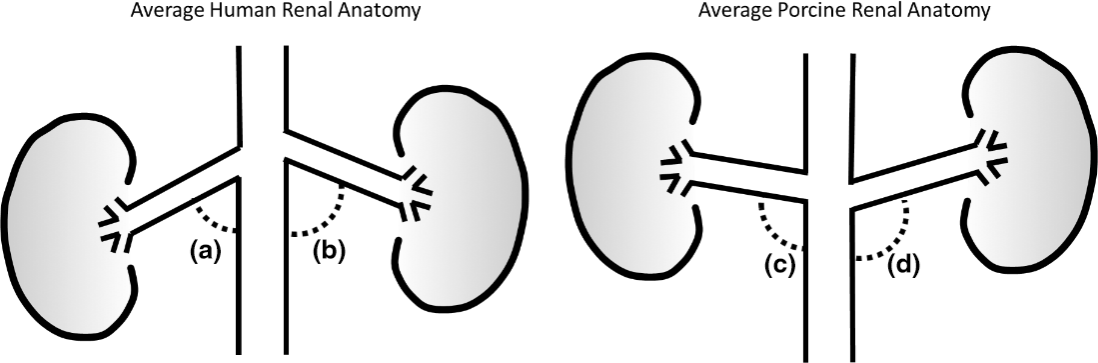
Schematic representation of the average renal artery takeoff angle derived from a postmortem study in humans (Wozniak, 2000) compared with the average takeoff angle observed in this study utilizing porcine anatomy; Angle A = 75°, Angle B = 85°, Angle C = 101°, Angle D = 110°.

After gathering and analyzing the porcine renal angiograms, there were several findings that were worth noting. To start, one finding was that all porcine main renal arteries measured in this study were straight, while at least half of all human renal arteries measured in other studies have shown to be tortuous (Hegedus, 1972; Mishall, 2016; Yagoub et al., 2022). This was done by qualitative observations of the renal angiograms like those shown in Figures 2, 3, 4. Additionally, the renal takeoff angles observed in these animals tend to branch off superiorly from the aorta, while studies have shown that human renal takeoff angles tend to branch inferiorly (Trani et al., 2009; Wozniak, 2000) as shown in Figure 7. Furthermore, measurements of the main renal arteries showed that despite having slightly smaller average diameters (1 mm and 0.5 mm at most in the left and right renal main respectively), the average lengths of the renal main arteries were comparable (LMA/RMA of 32.18 ± 7.46/42.23 ± 7.33 mm in porcine and 34.8 ± 12.5/41.4 ± 15.0 mm in human) (Lauder et al., 2018).

Upon perfusion of a the renal blocks, there were notable differences in the produced urine output rates between the fresh and frozen specimens. Although initial rates (*t* = 0) began at similar values, 450–500 mL/h, the average frozen specimen's outflow rates tended to be about 10× greater than the rates of the fresh specimens. Despite the magnitude differences between the two different samples, flow trends throughout the 3‐h study remained the same. After initial perfusion, there was an increase in urine output rates which peaked at the 45‐min time mark in both specimens, followed by a steady decline. We believe the decline after the 45‐min time point to be a result of edema, which was observed using the overhead cameras, which could have led to a decrease in buffer circulation through the isolated tissue and thus reduced urine output. Interestingly, the urine output rates of all fresh specimens, throughout the 3‐h study period, were above the criteria for kidney risk, injury, or failure based on the RIFLE, AKIN, and KDIGO scoring (Bellomo et al., 2004; Disease, 2012; Mehta et al., 2007). Future studies should be performed in which the study protocol is extended as needed to determine how long a renal block would function on the Visible Kidney™ apparatus before renal risk, injury, and/or failure is observed.

Additionally, the urine analyses showed that all averaged biomarkers measured were statistically different between the fresh and frozen renal blocks. These biomarker averages over time also show differences in trends between specimens when compared to the Krebs composition. When looking at the pH, sodium (Na), potassium (K), and glucose concentrations, the frozen specimens had averages across the study duration closer to that of the overall Kreb's buffer concentrations when compared to the fresh specimens. This likely indicates that the frozen specimens had little to no tubular reabsorption of these electrolytes when compared to the fresh specimens. However, when observing calcium concentrations, the frozen specimen averages indicate a higher retention of calcium. Further work is needed to better understand the role of calcium secretion/absorption using this setup. Also worth noting, we believe the high level of potassium observed in the fresh tissue at *t* = 0 to be from the cardioplegia solution that is administered to induce cardiac arrest before the hearts and renal blocks were procured. These levels then normalized over time once the excess potassium was filtered out after perfusion of the kidney block began.

While the refinements of our IPK model are not complete and will not fully replace the use of animals, this approach has the potential to reduce the number of animals needed for research objectives. More specifically, we have begun to utilize this preclinical research tool to investigate medical technologies utilized for renal denervation (RDN) therapies. Figure 4 highlights the use of the Symplicity Spyral™ system in our IPK model. With the latest data of the SPYRAL HTN‐ON MED (MD Kandzari et al., 2018) showing safety and efficacy of catheter‐based renal artery denervation and the long‐term follow‐up of the SYMPLICITY HTN‐3 Trial (Bhatt et al., 2022), we anticipate a significant growth in the RDN therapy area and increased need for preclinical models mimicking human use conditions. Such models will be critical in the design, development (Coates et al., 2022), teaching, and adoption of this therapy and its next‐generation devices.

**FIGURE 8:**
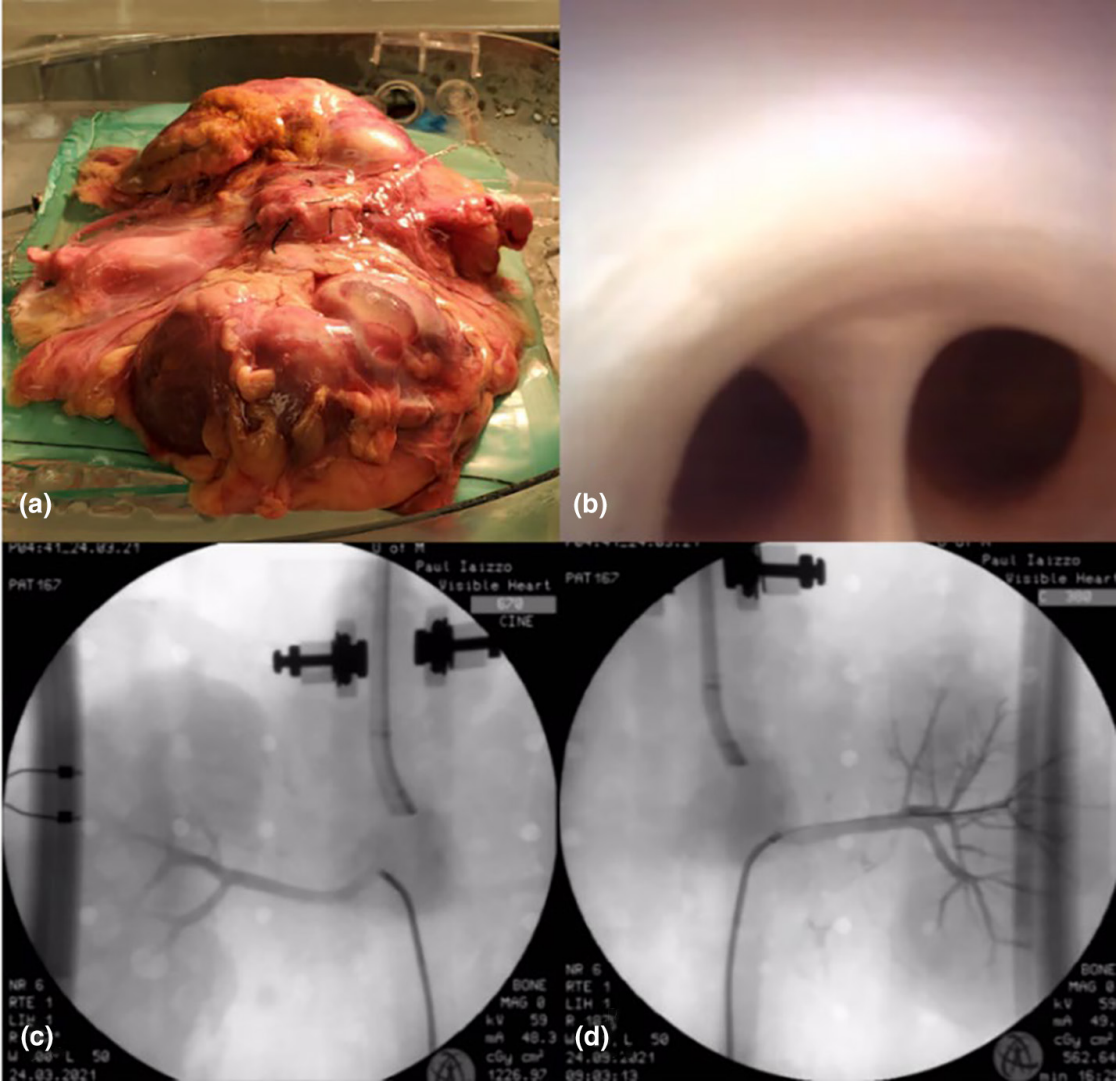
(a) After being extracted and prepped using the same methods as the isolated porcine model, the human kidney block was positioned and perfused. (b) Next, endoscopic cameras were inserted to visualize the internal anatomy. Here, we can see the main bifurcation of the right renal artery. Multimodal images of human renal arteries were acquired with fluoroscopy, allowing for the capture of renal angiograms of the (c) right and (d) left renal arteries.

Just as the Visible Heart® Laboratories receives human donor heart‐lung blocks for research, so too can we plan to use human donor kidneys. To date, we have been able to isolate from a fresh human cadaver, that was CT imaged beforehand, a renal en bloc for placement in the apparatus. This specimen was extracted and prepared using the same methods as described in the porcine model. Once prepared, the human renal block (*n* = 1) was perfused and utilized just as the porcine model, Figure 8, while taking the necessary biohazard precautions.

### 4.1 Limitations

While we consider this preclinical benchtop model to be an improvement over several pre‐existing IPK models, it is not without its limitations. Though this approach is meant to simulate human use conditions, porcine anatomy was primarily used for this research. In general, the porcine organs used in these studies had smaller diameters and more superior renal artery takeoff angles when compared to the average human (Lauder et al., 2018; Trani et al., 2009; Wozniak, 2000). Additionally, the results presented here indicate that porcine main renal arteries are less tortuous than humans via qualitative analysis and future studies can be done to quantify these differences. However, the characterization of these characteristics allows researchers to account for these differences while using this model.

When employing this approach utilizing human donor kidneys, on the Visible Kidney™ apparatus, the compliancy and nature of the tissues and vasculatures relative to their disease states, if existing, should remain the same as when ex vivo; further studies are needed to confirm as such. Furthermore, the uses of healthy swine anatomies are much more compliant and may not be representative of that of diseased human anatomy. While external parameters on the Visible Kidney™ can be altered to mimic disease states, such as decreasing blood flow or increasing pressure to simulate atherosclerosis or hypertension respectively, using diseased donor tissues will still the best way to truly recreate diseased states in our IPK model.

Here, the initial chemical analysis performed during these studies could be further expanded to evaluate additional biomarkers that may more directly correlate to renal function. One such biomarker we have begun to implement is creatinine, which can be added to the Krebs buffer solution; used to directly assess glomerular filtration rates (GFRs). Another limitation in this study is the use of the Krebs buffer which had a lower viscosity (0.79 ± 0.20 cP) when compared to blood (3.5–5.5 cP). These differences in viscosities could likely be associated with higher‐than‐normal urine output rates (Schinstock et al., 2021) and would therefore warrant additional studies with varying buffer viscosities to confirm this relationship.

## 5 CONCLUSION

An improvement to pre‐existing isolated perfused kidney models was achieved through the refinement of the organ procurement process and the development of an apparatus that more accurately recreates human physiologic use conditions. By simulating the microenvironment of the abdominal cavity by controlling temperatures, pressures, flows, and pump rates, the isolated renal blocks were shown to elicit relative viabilities for up to 3 h after reperfusion. Because of its anatomic and physiologic similarities, the use of viable porcine (or any large mammal) renal blocks in this preclinical model can aid to refine, reduce, and in some cases replace additional large animal studies, while providing a setting in which advancements in renal pharmacology and/or medical devices can be made. While the Visible Kidney™ model is being further developed to address the limitations presented in this work, the specific methodologies behind the design are currently patent pending; such an approach should be helpful for medical device innovations, training, and education in general.

## CONFLICT OF INTEREST STATEMENT

Thomas Valenzuela, Emma Schinstock, Samantha Kohnle, and Stefan Tunev are employees at Medtronic Inc. The Medtronic and University of Minnesota co‐authors also have a patent pending for the invention entitled “Perfused isolated large mammalian organ apparatus which mimics a variety of physiologic conditions for the development, testing and assessment of therapies/devices, procedural efficacy, nerve viability and organ function (e.g., Kidney, Liver, Spleen).” Dr. Latib is a consultant for Medtronic, Abbott, Edwards Lifesciences, Boston Scientific, and Philips. Dr. Bliagos has no disclosures to report. Paul Iaizzo is a Professor at the University of Minnesota and has a research contract with Medtronic.

## ETHICS STATEMENT

The original protocol was reviewed by the Institutional Animal Care and Use Committee at the University of Minnesota. However, since these studies utilized what would have otherwise been waste tissue, the University of Minnesota Institutional Review Board waives review and approval of research using waste tissue.
